# A Simple Distance Paper-Based Analytical Device for the Screening of Lead in Food Matrices

**DOI:** 10.3390/bios11030090

**Published:** 2021-03-22

**Authors:** Kasinee Katelakha, Vanida Nopponpunth, Watcharee Boonlue, Wanida Laiwattanapaisal

**Affiliations:** 1 Interdisciplinary Program of Biomedical Sciences, Graduate School, Chulalongkorn University, Bangkok 10330, Thailand; kkasinee.hsc@gmail.com; 2 Department of Clinical Chemistry, Faculty of Allied Health Sciences, Chulalongkorn University, Bangkok 10330, Thailand; Vanida.N@chula.ac.th; 3 The Halal Science Center, Chulalongkorn University, Bangkok 10330, Thailand; 4 Department of Nutrition and Dietetics, Faculty of Allied Health Sciences, Chulalongkorn University, Bangkok 10330, Thailand; Watcharee.Bo@chula.ac.th; 5 Biosensors and Bioanalytical Technology for Cells and Innovative Testing Device Research Unit, Chulalongkorn University, Bangkok 10330, Thailand

**Keywords:** distance paper-based device, carminic acid, polyethyleneimine, lead detection, standard addition, food matrices

## Abstract

A simple and rapid distance paper-based analytical device (dPAD) for the detection of lead (Pb) in foods is proposed herein. The assay principle is based on competitive binding between carminic acid (CA) and polyethyleneimine (PEI) to Pb in a food sample. The paper channels were pre-immobilized with PEI, before reacting with a mixture of the sample and CA. Pb can strongly bind to the CA; hence, the length of the red color deposition on the flow channel decreased as a lower amount of free CA bound to PEI. The dPAD exhibited good linear correlation, with ranges of 5–100 µg·mL^−1^ (*R*^2^ = 0.974) of Pb. Although, the limit of detection (LOD) of this platform was rather high, at 12.3 µg·mL^−1^, a series of standard additions (8.0, 9.0, and 10.0 µg·mL^−1^) can be used to interpret the cutoff of Pb concentrations at higher or lower than 2 µg·mL^−1^. The presence of common metal ions such as calcium, magnesium, nickel, and zinc did not interfere with the color distance readout. The validity of the developed dPAD was demonstrated by its applicability to screen the contamination of Pb in century egg samples. The results obtained from the dPAD are in accordance with the concentration measured by atomic absorption spectroscopy (AAS) (*n* = 9). In conclusion, this proposed dPAD, combined with the standard addition method, could be applied for screening Pb contamination in food matrices. This platform is, therefore, potentially applicable for field measurements of Pb in developing countries, because it is cheap and rapid, and it requires no significant laborious instruments.

## 1. Introduction

Century egg, otherwise known as preserved egg or Pidan, is one of the famous Chinese and Thai cuisine ingredients because of its unique taste, high nutrition, and affordability. In the manufacturing process, there is likely the misuse of lead oxide, with it being added into the pickling mixture in order to reduce the ripening time and also to help improve the texture of the century eggs. However, the lead (Pb) content in the pickling mixture could also permeate through the eggshells and egg membrane into all parts of the eggs, thereby accumulating in the egg whites and yolks [1,2]. Hence, the Pb content in century eggs has become a major safety concern, because food consumption is a major pathway of toxic compounds for human beings [3]. Pb is indicated as a neurotoxic element, which could be the cause of long-term serious health problems. Accumulation of Pb in the human body could damage not only the nervous system, but also the immune system [4]. The permissible content of Pb in century eggs regulated by China’s food safety standards is less than 2 µg·mL^−1^ [3] which corresponds to the value permissible by the Food and Drug Administration (FDA) of Thailand [5].

The existing reliable methods that can be used for the quantitation of Pb are atomic absorption spectroscopy (AAS), inductively coupled plasma optical emission spectrometry (ICP/AES), and inductively coupled plasma mass spectroscopy (ICPM) [6]. The major advantage of these methods is their high sensitivity, as they can detect analytes at very low concentrations with exceptional reproducibility. However, those methods are expensive and time-consuming, and they require well-trained personnel. Therefore, chemical and biological sensing technologies have been considered as alternatives for the rapid and low-cost detection of Pb [7,8]. To achieve sensitivity and to improve selectivity for the detection of Pb and other heavy metals simultaneously, several strategies have been proposed. These methods are based on K^+^-induced specific DNA G-quadruplexes [9], gold nanoparticles (AuNPs) [10], and DNAzymes [11]. However, these methods are based on the assistance of instruments. Nevertheless, for methods of on-site determination, users are attracted not only because of their reliable platforms, but also because they are lightweight and cheap, and they offer rapid detection. The paper-based assay method is, thus, attracting considerable attention as a matrix for fabricating a point-of-care device that is lightweight and highly abundant. Moreover, paper is made from cellulose fibers with a high volume-to-surface ratio and permits the transport of fluids via capillary forces without requiring external pumping equipment.

The colorimetric measurement of Pb using paper-based devices has already been established, in which the method is based on the reduction of silver nitrate (AgNO_3_) using sodium borohydride (NaBH_4_) as a reducing agent and polyvinyl alcohol (PVA) as a capping agent. In the presence of Pb, the aggregation of AgNO_3_ results in a reddish-yellow color that can be observed and quantitated using a smartphone and ImageJ software [12]. Generally, the quantitation of the color intensity on the paper can be complicated by some factors, including different color perceptions and lighting effects. These factors reduce the sensitivity and reproducibility of an assay. Therefore, this assay method usually requires an external paper scanner and specific programs for image analysis.

The distance paper-based analytical device (dPAD) has advantages over a colorimetric assay because it does not require any extra instruments for measurement and quantitation. The measurement is simple, requiring only a readout of the color distance from the nearby ruler. Distance-based detection is one of the promising semi-quantitative analysis methods with superiority to the colorimetric detection-based method [13,14,15,16,17,18]. Several approaches have been proposed for improving the performance of the dPADs, such as using organic solvent to enforce the opening of wax valves, which facilitates reagent mixing and incubation in distance paper-based analytical devices [19]. The wax printing of a microchannel of a dPAD platform on a plastic substrate could accomplish a reduction of the volume of an analyte [20]. The distance-based approach can also be performed on a cotton thread substrate, which is an alternative material to paper for the fabrication of the fluidic sensor for the analysis of environmental contaminants [21,22]. In addition, by combination with a pre-concentration approach, such as headspace microextraction [23], solid-phase extraction [24], and a portable heater [25], the sensitivity of measurements can be improved. Furthermore, there are several reports of using distance-based applications for clinical diagnosis such as chloride test [26], potassium [27], alkaline phosphatase [28], and cardiac troponin I [29]. For the detection of metal ions, the dPADs have also been previously reported using the principle based on the deposition of chemical chelating compounds, such as dithizone [30] and a synthesized porphyrin [31]. Interestingly, the growing interest in dPAD application is reflected by the increasing number of publications during recent years, by using several strategies such as G-quadruplex-DNAzyme [32], starch hydrolysis [33], ionophore-doped ion-selective nanospheres [34,35], and synthesized fluorescent ligands [25,36].

In this research, we propose a distance-based detection method for Pb-contaminated food matrices using century eggs as an example. The potential chelating agent used was carminic acid (CA). It is generally known that CA is red in color, which is produced by insects, and it has been used historically as a natural food dye because it is a less toxic substance. Moreover, there are previous reports of a complex formation between CA and Pb in buffer solutions [37,38]. The color of the solution changes from red to purple when Pb is present, as can be detected by a spectrophotometer. However, the color development when CA is deposited directly along a hydrophilic channel on paper cannot be observed because of color fading due to the exposure of CA to light and oxygen [39]. Moreover, the negative functional group of CA molecules does not promote chemical interaction between CA and cellulose paper during the reaction. The dispersion of dye in porous paper leads to a faint and milder color on white paper. To overcome such a problem, herein, the paper surface was modified with positively charged molecules, i.e., polyethyleneimine (PEI), to entrap the dyes to enhance the color intensity on paper, as this approach has been successfully applied previously for entrapping unbound hydroxynapthol blue as a loop-mediated isothermal amplification (LAMP) solution proposed by our group [40]. PEI is composed of repeating units of amine groups and a CH_2_CH_2_ spacer, and it possesses strong binding capacity to negatively charged compounds. PEI has been successfully used to modify the paper surface to quantify the initial concentration of genomic DNA [40].

In this article, we propose an indirect approach for the screening of low concentrations of Pb in food matrices using a dPAD. PEI is an imperative immobilizing agent in the flow channels of porous paper, allowing to trap free negatively charged dyes and CA, thereby forming a color distance readout. In this study, the measurement was based on the distance given by free CA bound to immobilized PEI on paper after forming a complex with Pb in the solution. As such, a shorter distance implies a higher Pb concentration. To improve the sensitivity of our proposed method to ensure its efficiency for the detection of Pb contamination in food samples, a series of Pb concentrations were added to a sample and performed in parallel on the same device. A straight line was obtained by connecting the top of the color distance of the serial concentrations of Pb to see the trend of the curve, in which the Pb concentration could be determined through the specified color index, as this approach was inspired by the method for screening the albumin-to-creatinine ratio described by Hiraoka et al. [41]. By using this approach, the dPAD reported herein can be used to determine Pb contamination at the cutoff level for food safety control for century eggs. Furthermore, it is economically and environmentally friendly, and it is superior to other conventional methods because it is easy to operate, it is portable, and it does not require any external instruments. Our proposed method provides great promise for quick decision-making regarding food safety concerns because of a standalone and read-by-eye detection method.

## 2. Materials and Methods

### 2.1. Chemicals and Reagents

AAS standard grades of Pb, Ca, Mg, Cu, Ni, and Zn at concentrations of 1000 mg·L^−1^ in 3% nitric solution were purchased from Merck KGaA (Darmstadt, Germany). Hydroxyethyl piperazineethanesulfonic acid (HEPES) of Vetec^TM^ reagent grade, branched PEI with an average molecular weight of 25 kDa, and CA were purchased from Sigma-Aldrich (Sigma-Aldrich, St. Louis, MO, USA). A buffer composition, including sodium dihydrogen phosphate and Tris(hydroxymethyl)aminomethane, was obtained from Sigma-Aldrich (Sigma-Aldrich, St. Louis, MO, USA). Analytical reagent grade sodium hydrogen diphosphate, potassium cyanide, and sodium acetate were purchased from Ajax Finehem (Sydney, Australia). American Chemical Society (ACS) grade acetic acid (glacial) and hydrochloric acid fuming 37% were purchased from Merck KGaA (Darmstadt, Germany). All buffer solutions were prepared in ultrapure (UP) water with a resistivity of 18.2 MΩ·cm (Thermo Fisher Scientific, Abingdon, UK). Whatman^TM^ filter paper, Grade 1004 (No. 4), was purchased from GE Healthcare Life Sciences (Hatfield, UK). A wax printer was employed for paper fabrication (Fuji Xerox, ColorQube8870, Tokyo, Japan). The Multiskan GO^TM^ microplate reader (Thermo Fisher Scientific, UK) was used for spectrophotometric studies. Fourier-transform infrared (FT-IR) analysis was archived by FT-IR Tensor ll of Bruker (Karlsruhe, Germany). SEM images were collected using SEM Quanta 250 (FEI^TM^, Hillsboro, OR, USA). AAS was analyzed by AGILENT 280 FS at the Scientific and Technological Research Equipment Centre (STREC), Chulalongkorn University, Thailand.

### 2.2. Design and Fabrication of the dPAD

**Figure 1:**
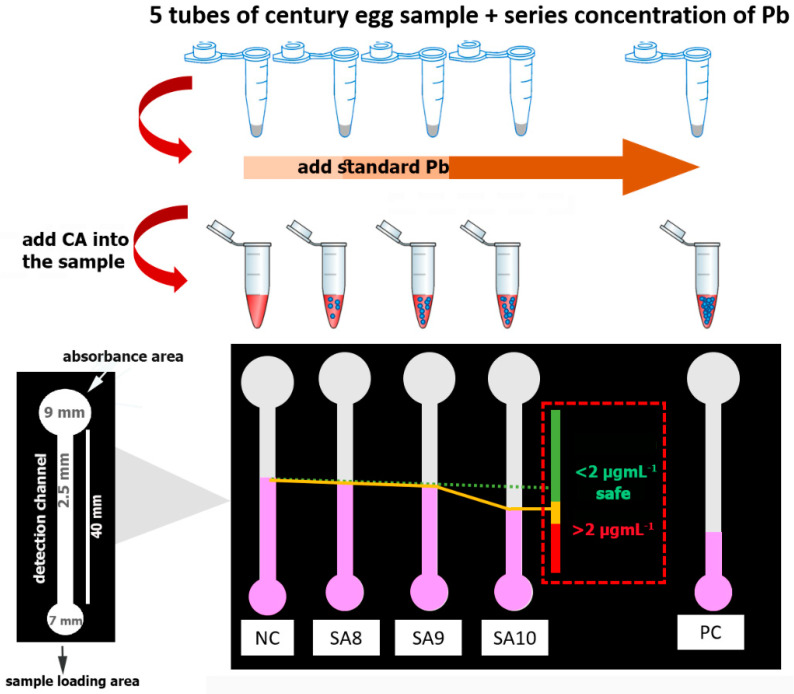
Demonstration of the screening analysis of lead (Pb) in century egg samples using the developed distance paper-based analytical device (dPAD) combined with the standard addition technique for evaluation at the cutoff concentration of Pb. CA, carminic acid. NC, negative control; SA8, SA9, and SA10, sample with standard Pb addition at the concentration of 8, 9, and 10 µg·mL^−1^, respectively; PC, positive control.

The dPAD was designed using the Microsoft Office PowerPoint 2010 program. The designed pattern was printed onto filter paper using the wax printing technique [42] to create hydrophilic and hydrophobic barriers. In our design, as depicted in Figure 1, a sheet of paper composed of five channels was used for one sample measurement. With each channel, a circular shape with a diameter of 7.0 mm was created for the sample loading area. The circular shape was connected to a straight hydrophilic channel with a width of 2.5 mm and a length of 40 mm and was used as the detection area. A larger circular shape (diameter of 9.0 mm) was designed on top of the detection channel to assist fluid flow by capillary action along the straight hydrophilic channel. After wax printing, the designed dPAD was heated to 150 °C for 2 min to melt the wax and to allow it to penetrate through the thickness of the membrane paper, as well as to create hydrophobic barriers to manipulate the fluid flow. After that, the wax-printed paper was attached by double-sided adhesive tape to a plastic transparent sheet in order to strengthen the device and to control the flow during the analysis.

### 2.3. Modification of the Membrane Surface by PEI

The hydrophilic paper channels were modified by immobilizing the PEI to improve the absorption efficiency of CA on the dPAD. In this study, the effect of PEI on the color distance of CA was studied at PEI 0.02% and 0.05%. The aqueous solution of PEI was prepared in UP water. Then, the aqueous solution of PEI (7.5 µL) was immobilized along the paper channels using the manual pipetting technique. After that, the channels were allowed to dry at room temperature. The PEI-immobilized dPAD was kept under dry conditions using a desiccator.

To find a suitable concentration of PEI, the CA solution was dissolved in 0.1 M HEPES buffer (pH 7.0) to gain a final concentration of 0.0–2.0 mmol·L^–1^. A solution of 20 μL of CA solution was pipetted into the sample loading area of the dPAD and left to wick along the detection channel until the solution reached the top of the dPAD. The concentration of PEI able to entrap CA, but not too high to block the wicking solution, was selected as an appropriate concentration. In addition, the interaction of PEI and CA on the membrane was further characterized with SEM analysis.

### 2.4. The Assay Principle

The assay principle refers to integrating the binding property of CA to Pb under appropriate pH conditions. The CA–Pb complex could be trapped in the sample loading area, allowing only free CA to wick through the hydrophilic detection channel, entrapped by the pre-impregnated PEI. In brief, the reagent buffer (0.4 mmol·L^−1^ CA solution) was mixed with the analyte sample at a 1:1 (*v*/*v*) ratio and incubated for 5 min at room temperature. After mixing, the solution mixture (20 µL) was loaded into the sample loading area of the dPAD. The color distance was read when the solution reached the top of the platform. Therefore, the color distance of CA visualized from the detection channel was reduced when presented with high concentrations of Pb. The formation of the color distance was easily observed on the immobilized detection channel of the dPAD. The color distance could be measured with a Vernier scale meter to obtain two-digit accuracy. Additionally, the screening of Pb in the sample could be estimated using the naked eye after drawing a line and interpretation together with the standard addition method described in Section 2.5.

### 2.5. Real Sample Analysis

To demonstrate the applicability of our proposed dPAD for the screening of Pb-contaminated food matrices, century eggs spiked with Pb were used as tested samples. UP water was added to the whites (50 g) of the century eggs to gain a 1:1 (*w/v*) ratio and it was homogenized using a blender. Then, the supernatant was filtered through a Whatman No. 1 membrane filter with the assistance of a peristaltic pump. The filtrated supernatant of the sample was divided into nine portions and spiked with Pb to obtain a final concentration ranging from 0 µg·mL^−1^ to 3 µg·mL^−1^, which were used as blind samples (*n* = 9). Then, the Pb level in the blind samples was determined with the proposed dPAD using the protocol of the standard addition technique. In brief, the food samples (50 µL) were aliquoted into five microcentrifuge tubes, to which the standard Pb was added to gain final concentrations of 0.0, 8.0, 9.0, 10.0, and 40.0 µg·mL^−1^. Herein, samples with 0 and 40 µg·mL^−1^ Pb were used as a negative and positive control, respectively. The reagent buffer (0.4 mmol·L^−1^ CA solution in 0.1 M HEPES buffer at pH 7.0) was added to each microcentrifuge tube at the ratio of 1:1 (*v*/*v*) and mixed thoroughly, before incubating for 5 min at room temperature to allow the binding of CA and Pb. Then, 20 µL of the solution mixture was loaded into each channel of the PEI-immobilized dPAD. The red color distances from each channel were observed, and a line was drawn from low to high concentrations. The level of Pb in the food samples was determined by simply observing the straight line connecting the top of a series concentrations through the color identification chart, as demonstrated in Figure 1. The validity of the dPAD for Pb detection was confirmed by the AAS standard method.

## 3. Results

### 3.1. Distance-Based Detection Method

The potential of using CA for the rapid screening of Pb with the approach of distance-based color formation by the CA–Pb complex was investigated. A preliminary study was performed using CA immobilized directly onto the designed detection channels of the dPAD. However, red color formation along the channels was not observed as expected. The color fading might be due to the penetration of CA color through the porous membrane. Moreover, it was found that CA could be moved to the top of the dPAD when the solution was applied, which implies that the CA did not strongly adsorb into the paper channels. Therefore, it was assumed that it is not possible to directly apply CA to the dPAD to perform color distance detection of Pb. Therefore, the hydrophilic channel needed additional modification by a cationic polymer to enhance the binding with free CA and generating a color distance. In our study, the strong positively charged polymer PEI was used for modification of the paper surface.

The suitable concentration of PEI for immobilizing the detection channel of the dPAD was investigated. The PEI solution was prepared by dissolving it in ultrapure water. The results demonstrate that PEI at the concentration of 0.1% could block the membrane pores, leading to CA not being able to move along the detection channel. Therefore, in this study, PEI was then diluted to gain final concentrations of 0.02% and 0.05% (*w*/*v*). Various concentrations of CA were loaded into the sample loading area and allowed to travel along the PEI-immobilized detection channels. It was found that the color distance increased as the CA concentration increased. A saturation curve was found when the concentration of CA reached 2.0 mmol·L^−1^. Linearity was observed in a range of 0.2–1.0 mmol·L^−1^ in both PEI concentrations, as shown in Figure S1 (Supplementary Materials). It was found that PEI at the concentration of 0.02% afforded a 1.6-fold higher sensitivity than that obtained from 0.05% PEI. In addition, using PEI at 0.02% immobilization, the color distance observed on the PEI-immobilized channel exhibited color stability, as the color distance precipitated on the channels did not fade with time. Thus, it could be suggested that the CA color stabilized by forming a complex with PEI on the dPAD. Unless otherwise stated, our dPAD was prepared using Whatman membrane No. 4 and the channels were pre-immobilized with 0.02% PEI before analysis.

### 3.2. Effect of pH

pH affects the net charge of PEI, thereby influencing the binding of PEI to CA. Therefore, various buffer and pH values, including sodium acetate buffer (pH 6.0), HEPES buffer (pH 6.0 and 7.0), sodium phosphate buffer (pH 6.0, 7.0, and 8.0), and Tris-HCl (pH 8.0 and 9.0), were investigated. It was found that the acidic buffer gave the shortest color distance, relying on the stronger interaction between PEI and CA (see Figure S2, Supplementary Materials). The color distances measured from CA dissolved in alkaline buffer were longer than those observed for the acidic buffer. Interestingly, no binding interaction was observed between PEI and CA when using phosphate buffer, whether under acidic or basic pH. Using phosphate buffer, the CA color moved through the PEI-immobilized detection channel without absorption or precipitation. The precipitation of a color distance measured when using CA prepared in phosphate buffer was, thus, not detectable. Thus, HEPES buffer, which is an ampholyte from a zwitterion structure, provided a broad range of buffering capacity of an environmentally relevant pH region, in addition to biological medium compatibility. A further advantage of HEPES is that it is a compound, whereby the binding constant to metal ions is negligible. Therefore, HEPES buffer (pH 7.0) was selected as an appropriation buffer solution for this method.

### 3.3. SEM Analysis

**Figure 2:**
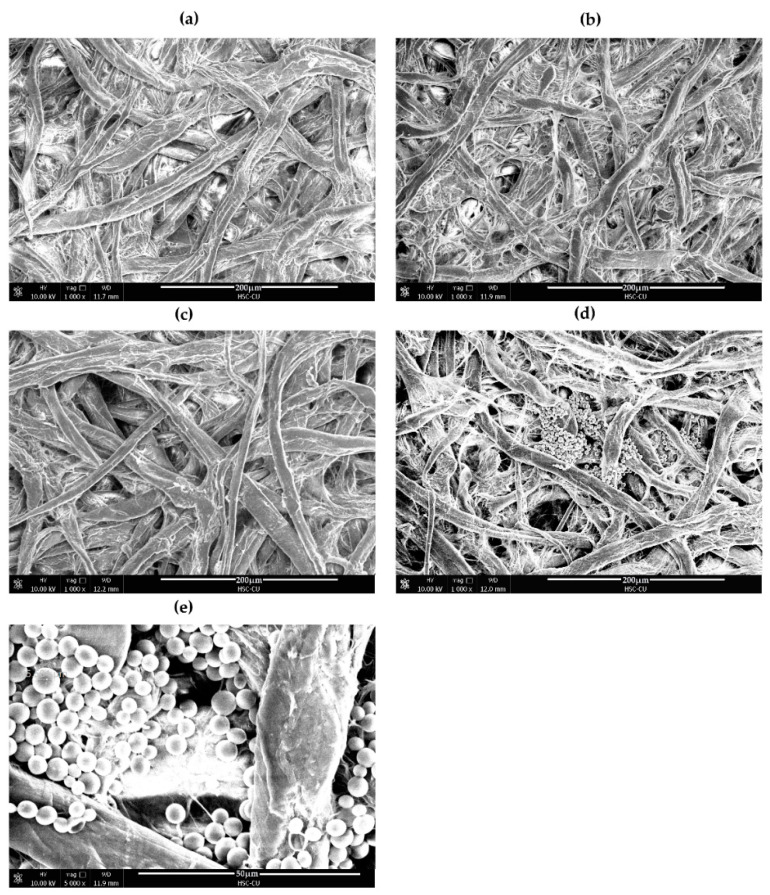
(**a**) Bare Whatman grade 1004; (**b**) polyethyleneimine (PEI) immobilized on Whatman filter; (**c**) CA immobilized on Whatman filter; (**d**) PEI–CA complex on Whatman filter (1000×); (**e**) PEI–CA complex on Whatman filter (5000×).

The complex formation between PEI and CA on the membrane of dPAD was investigated with SEM. Various conditions were modified on the bare cellulose-based membrane to see surface morphology changes, as shown in Figure 2. Compared to the unmodified cellulose membrane in Figure 2a, dense surface morphologies were found when a surface membrane was modified by PEI (Figure 2b), suggesting that the PEI was successfully coated onto the membrane surface. However, the membrane pores were still noticeable. On the contrary, the surface of the unmodified membrane with deposition of the CA solution was no different to the bare membrane (Figure 2c). This means that the CA did not interact with the paper cellulose fiber. Interestingly, particles with spherical morphology were clearly observed when the CA solution was added to the PEI-modified surface membrane (Figure 2d,e). This suggests that these distinctive circular structures are indicative of the condensation of highly positively charged PEI with negatively charged CA, forming stable spherical shape particles with sizes ranging from 1 to 5 microns. The condensation of PEI with CA resulted in visual detection on the paper membrane. This condensed spherical structure is rather similar to that of PEI complexation with DNA, which is very useful for gene delivery systems, as well as for the protection of DNA from enzyme degradation [43].

### 3.4. Measurement of Pb on the dPAD

**Figure 3:**
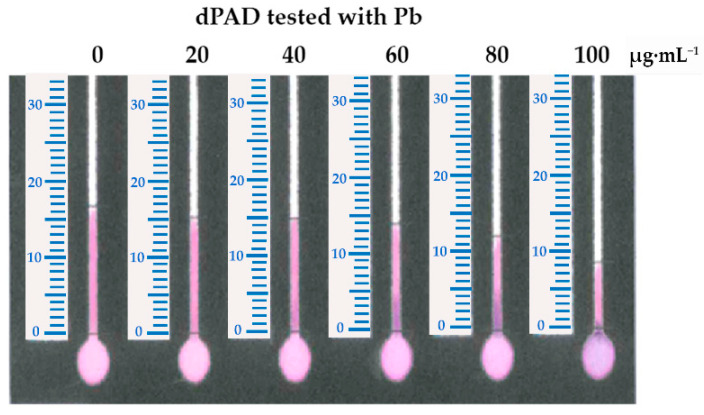
The decrease in the color distance on the PEI-immobilized dPAD observed when introducing Pb at concentrations ranging from 0 to 100 µg·mL^−1^ with 0.8 mmol·L^−1^ CA in 0.1 M hydroxyethyl piperazineethanesulfonic acid (HEPES) buffer (pH 7.0). The reaction was performed at room temperature for 5 min of incubation.

**Figure 4:**
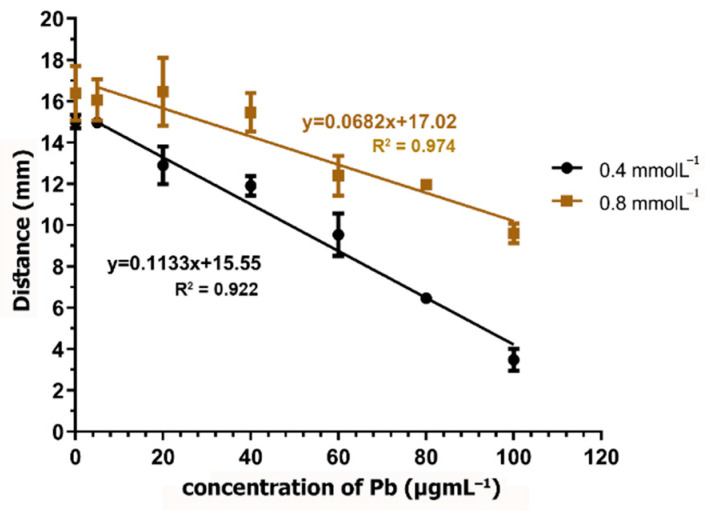
Color distance reduction when increasing the concentration of Pb using 0.4 and 0.8 mmol·L^−1^ CA, with the Pb concentration ranging from 0 to 100 µg·mL^−1^ in 0.1 M HEPES buffer (pH 7.0). Error bars represent triplicate measurements.

The reaction of the CA and Pb complex was prepared using CA at a concentration of 0.8 mmol·L^−1^ in 0.1 M HEPES buffer (pH 7.0). The reduction in color distance when the Pb concentration was increased is demonstrated in Figure 3. Then, a suitable concentration of CA was determined by testing the concentrations of 0.8 and 0.4 mmol·L^−1^. It was found that, when using CA at the concentration of 0.4 mmol·L^−1^, a higher sensitivity was obtained when comparing the slope of the linear regression with that that observed when using CA at the concentration of 0.8 mmol·L^−1^ (Figure 4). In addition, using 0.4 mmol·L^−1^ CA, the analytical range of the measurement was found to be broader when the Pb concentration ranged from 5 µg·mL^−1^ to 100 µg·mL^−1^. Therefore, CA at the concentration of 0.4 mmol·L^−1^ was selected for further study. Using 0.8 mmol·L^−1^ of CA, the excess dye might interfere with the distance formation, as a faint red color was observed within the channel, especially at low Pb concentrations. Therefore, the color distance measured at 20 µg·mL^−1^ was not different from that at 0 µg·mL^−1^. According to the condition of using 0.4 mmol·L^−1^ of CA, the limit of detection (LOD) (3 SD/slope) was calculated as 12.28 µg·mL^−1^. Although the LOD of this method is higher than the permissible level of lead in food, using standard addition methods can report the existing lead contamination in real samples.

### 3.5. Interferences with Other Metal Ions

The selectivity of CA toward Pb was evaluated in the presence of other potential metal ions. The interference study was conducted using 20 µg·mL^−1^ Pb. Interfering ions, including Ca, Cu, Ni, Zn, and Mg, were prepared at a concentration ranging from 0 to 1000 µg·mL^−1^. The effect of these interfering ions was studied by introducing each of them to the Pb solution separately. The interference ratio was defined as the concentration ratio of the interfering ions that produced a ±5% signal change when presented with 20 µg·mL^−1^ Pb.

The color distance observed from the solution of Pb together with Ca, Ni, and Mg did not decrease significantly, even when their concentrations were increased to 20 times higher than that of Pb. The interference by Zn ions was observed as a color change from violet to red when its concentration was increased to 10 times higher than that of the 20 µg·mL^−1^ Pb signal. Therefore, the tolerance ratio of Pb to Zn calculated from the color distance of the dPAD was 10. However, interference by Cu was observed on the dPAD, in which it produced >5% change of the color distance when an equivalent concentration of Cu was added to the Pb test sample. In addition, the color intensity of CA significantly decreased in the test solution consisting of Cu. This suggests that Cu is able to bind with CA, similarly to Pb. In addition, the precipitated compound trapped upon the sample loading area was distinguished. Accordingly, there was no free CA wicking through the detection channel to perform a color distance when the Cu concentration was increased to higher than 120 µg·mL^−1^. Hence, a significant reduction in free CA molecules was also noticed from the significant reduction in the color distance on the dPAD (Table 1). The decrease in the Pb color distance was not only due to the binding property of both Cu and Pb toward CA, but also because of the oxidation of CA in the presence of Cu [44]. This can lead to the loss of color of CA, and it was, thus, not surprising that a decrease in Pb signal distance was dramatically observed. To demonstrate the reduction in the effect of Cu using the dPAD, potassium cyanide (KCN) was used as a masking agent. In this study, KCN was prepared at the concentration of 5% and was added to the testing solution comprising Pb and various concentrations of Cu. The percentage reduction was calculated by comparison with the color distance of Pb alone. It was found that, in the presence of KCN, the color distance reduction was deemed not to have decreased dramatically, as the same reduction was found previously when KCN was not introduced. The tolerance ratio for Pb to Cu was, thus, found to be better, as the concentration of Cu could be increased to 40 µg·mL^−1^ (see Figure S3, Supplementary Materials). Therefore, it could be concluded that the interfering effect of Cu can be eliminated by the addition of KCN as a masking reagent.

### 3.6. Proof of Concept for Analysis of Pb on the dPAD

**Figure 5:**
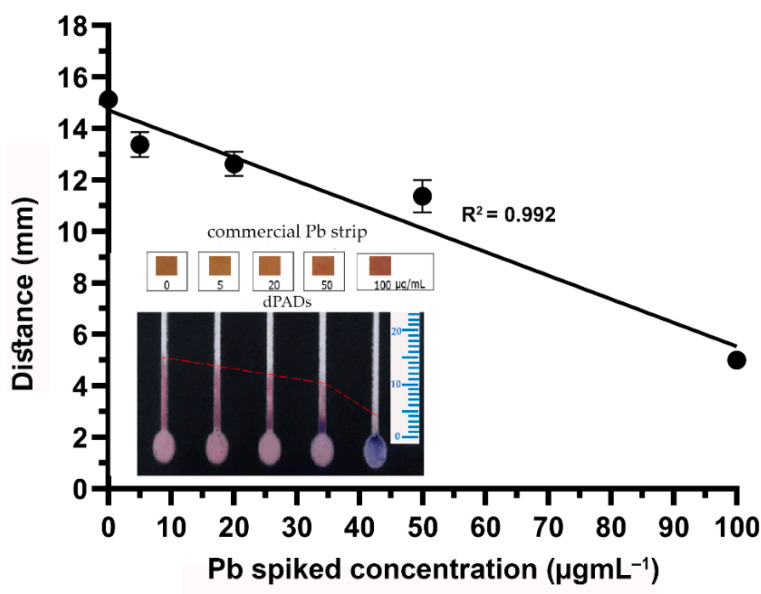
Drinking water with Pb (0–100 µg·mL^−1^) tested with commercial strips and the dPAD using 0.4 mmol·L^−1^ CA (*n* = 3).

To confirm that the developed dPAD was capable of detecting Pb, it was tested with drinking water obtained from a household water dispenser. However, Pb was not detectable because the sensitivity of the dPAD was not high enough. The drinking water was then spiked with standard Pb to gain final concentrations of 5.0, 20.0, 50.0, and 100.0 µg·mL^−1^. As shown in Figure 5, the reduction in color distance was related to the increasing concentration of Pb. In addition, precipitation of the CA–Pb complex could clearly be observed on the dPAD when testing with Pb at a concentration higher than 50.0 µg·mL^−1^ (Figure 5 insert). Therefore, it is believed that our proposed dPAD can be applied for the determination of Pb in real samples. In addition, colorimetric-based test strips commercially available on the market were also used for the determination of Pb in parallel with our dPAD. According to the package insert, the strips can provide a detection range of 0–500 µg·mL^−1^. The color changed from yellow to orange when the concentration of Pb was in a range of 0–100 µg·mL^−1^, while it turned to red-violet when the concentration of Pb increased to 500 µg·mL^−1^. The color changes of the commercial test strips could be observed rapidly within a minute. However, it was rather difficult to distinguish the yellow-brown color against a paper background color, particularly at low Pb concentrations. Therefore, there could be a source of error in the variation of individual color perceptions. In conclusion, as a proof of concept, dPADs fabricated using PEI-immobilized detection channels have the ability to screen for Pb on the basis of color distance visualization.

### 3.7. Real Food Sample Analysis

To implement the dPAD for the screening of Pb in food matrices, century eggs were used as a representative food sample because they are one of the most relevant pickling products associated with the high consumption of Pb. The supernatant obtained from the homogenized egg whites from the century egg samples was tested using the dPAD. However, the supernatant was more viscous than drinking water, because it was composed of abundant ovalbumin, other proteins, and lipids. In our study, we found that the viscosity of the supernatant can influence the color distance formation, as per the color distance reduction of ~14%–20% compared to that from drinking water. The precision of the developed method was determined at four Pb concentrations (0.0, 5.0, 20.0, and 40.0 µg·mL^−1^) spiked in the egg white supernatant.

To study intra-day and inter-day reproducibility, 153 pieces of dPADs were fabricated in the same batch and assayed with different spiked Pb concentrations. For the intra-day precision assay, each concentration was performed in 18 replicates. Color distances of 10.91 ± 0.68, 10.50 ± 0.54, 9.73 ± 0.49, and 6.25 ± 0.35 mm were observed. The intra-day precision was expressed as % coefficient of variations (CVs) of 6.28%, 5.16%, 2.67%, and 5.66%, when tested with spiked Pb at concentrations of 0.0, 5.0, 20.0, and 40.0 µg·mL^−1^, respectively (*n* = 72). Inter-day reproducibility was performed on three different days and each concentration was conducted in nine replicates. The color distances of the century egg spiked with 0.0, 10.0, and 40.0 µg·mL^−1^ were obtained at 10.84 ± 0.76, 9.96 ± 0.36, and 6.41 ± 0.55 mm, which could be expressed as %CVs of 7.05%, 3.66%, and 8.67%, respectively (*n* = 81). As the %CVs of both intra-day and inter-day reproducibility were less than 10%, the precision and reproducibility of our proposed dPADs were acceptable.

The standard addition technique is a well-known technique for minimizing the effect of sample matrices that interfere with the measurement of an analyte signal. To overcome the variation of the viscosity from different sample matrices, the standard addition method was exploited. Furthermore, the standard addition technique was also used to assist the dPAD’s performance for the screening of low concentrations of the target analytes. Different concentrations of Pb were added to the supernatant of the egg whites of the century egg samples; then, they were tested in parallel, together with negative and positive controls, using the same dPAD, as described in Section 2.4.

Blinded samples with Pb levels ranging from 0 µg·mL^−1^ to 3 µg·mL^−1^ (*n* = 9) were used to evaluate the developed dPAD. To discriminate samples with Pb levels over and below the cutoff permissible levels (2 µg·mL^−1^) according to the national standard, the concept of a drawing line interpretation was combined in our device. A straight line was drawn by connecting the top of the color distance of each channel. A reduction trend line could be distinguished on the basis of whether a sample contained Pb at higher or lower levels than the cutoff concentration. Therefore, the concentration of Pb measured under our proposed device could be expressed as Pb ≤ 2 µg·mL^−1^ and >2 µg·mL^−1^. These samples were also tested with the AAS standard technique to confirm the actual concentrations. The Pb levels determined with this proposed platform were in accordance with the concentrations measured by AAS. Only one sample (No. 3) was found to have ambiguous results. The dPAD expressed the concentration as ≥2 µg·mL^−1^, while, the actual concentration of Pb by AAS was 1.9568 µg·mL^−1^ (Table 2). However, the overestimation of 0.0432 µg·mL^−1^ could be due to the variations in color distance on the dPAD affecting the straight trend line, which is a possible source of error. Nevertheless, our purpose was to design a dPAD to use for screening purposes; thus, this was acceptable, since Pb contamination was not ruled out. Interestingly, all of the samples were also analyzed using the same commercial rapid test strips mentioned in Section 3.6. The color changes observed on the commercial strips were interpreted as “zero”, suggesting that all samples had Pb at lower levels than the kit’s detection limit, despite the detection range of 0–500 mg·L^−1^ claimed by in package insert.

## 4. Discussion

### 4.1. CA–PEI Complexation Using the dPAD Approach

The color fading limited the use of CA directly as a chemosensing agent for Pb under the distance-based strategy. The fading was caused by photobleaching of anthraquinone, which occurs due to the accumulation of tautomeric photoreactive triplet state exposed with oxygen [45]. Moreover, the color fading of anthraquinone dye could be attributed to emissions from tautomeric states following excited-state internal electron transfer and from the population of a dark, nonradiative state that irreversibly leads to a colorless molecule [46,47]. Therefore, the membrane surface of the dPAD needed further modification before interaction with the CA. Herein, the strategy of opposite charge interaction between negatively charged CA and strong positively charged PEI was utilized.

PEI is a polymer with a high concentration of amine groups in its structure. The amine groups provided a good environment for the formation of a CA–PEI complex, as reported in the literature [48]. The redshift spectrum of approximately 52 nm observed in our experiment could be described by the complex formation between PEI and CA (see Figure S4, Supplementary Materials). Additionally, the binding between PEI and CA could be described as an anthraquinone moiety of the CA-bearing anionic group interacting with the cationic hyperbranched structure of PEI [49]. According to our approach, the interaction between PEI and CA was reserved on the hydrophilic detection channel of the dPAD. A surface membrane pretreated with PEI for absorption of other dyes has previously been reported to improve the absorption efficiency [50]. The absorption performance in the hydrophilic channel of our dPAD could be explained in a similar manner. In comparison to these membrane modifications, bare membrane substrates could be used for the absorption of CA. However, it was observed that, after dropping the solution into the sample loading area, the impregnated CA was easily desorbed from the paper channel and moved toward the upper part of the channel. On the contrary, surprisingly, the PEI-modified membrane substrate was found to be complexed with CA with clear color formation along the paper channels. This was confirmed by the presence of spherical structures of PEI condensation on the paper surface when observed under SEM. This could be explained in a similar manner to that of PEI–DNA complexes [43,51,52,53]. Therefore, we assumed that the binding interaction of PEI and the anionic dye CA on the membrane substrate could take place in a comparable manner. In addition, the hydrophilicity property of the membrane after treatment with PEI remained. The binding interaction between PEI and CA on the membrane surface is, thus, promising for further application for the measurement of particular compounds by using the distance-based indirect strategy.

There are many factors that contributed to the color distance of our proposed dPAD. First, an excess concentration of PEI could have been trapped in the membrane of porous fibers [54], resulting in obstruction of the flow of CA. Therefore, using high concentrations of PEI for immobilization on the membrane can result in a shorter color distance formation. Second, the pH of the system contributed to the variation of the color distance measured on the PEI-immobilized channel, because it plays a major role in its electrostatic interaction with CA on the hydrophilic detection channel. A short color distance was observed from the acidic solution in comparison to the color distance under basic conditions. This can be explained because, under acidic conditions, the PEI chain is strongly positively charged due to the protonation of the secondary amine along the backbone chain of PEI [55]. In addition to the positive charge of PEI, the deprotonated forms of CA were also pH-dependent (pKa_1_ = 3.39; pKa_2_ = 5.78; pKa_3_ = 8.35 [56]), which could suggest that the deprotonated state of CA promotes the binding interaction of CA to PEI. Third, the detection channel’s width refers to the area available for the complex formation of PEI immobilized on the membrane and free CA [57]. This factor affects the slope of the linear regression line. In our experiment, a hydrophilic channel width of 2.5 mm was defined before wax printing. This width is workable, as suggested in the literature, as the channel width for the distance-based approach should not exceed 4.7 mm because the detection range will be unacceptably low [58].

### 4.2. Detection of Pb in Food Samples

The detection of Pb in food samples using the paper-based device is a challenge because it can be significantly influenced by the sample matrix. A bismuth-modified electrode fabricated by screen-printing on paper can increase the sensitivity of Pb detection for food and environmental analysis [59]. The paper-based assay was successfully implemented for selective detection of Pb in the environmental water using AgNPs/PVA as a plasmonic colorimetric probe, in which the color development relied on the aggregation of specific synthesized silver nanoparticles [12]. In addition, paper-based colorimetric detection has been proposed for Pb detection in a water sample, by using sodium rhodizonate as a colorimetric reagent for direct Pb detection on a PAD [60]. Nevertheless, most of the colorimetric detection on paper-based devices described previously relied on image analysis. However, simple and instrument-free detection by color visualization has not yet been reported for the detection of Pb in food matrices.

In this study, the developed dPAD proved promising for the screening of Pb in a complex food sample matrix. However, Cu showed partial interference with the color distance reduction. The interference by Cu is due to the complex formation between CA and Cu [56]; hence, false-positive results could have occurred. However, the interference by Cu can be diminished by adding KCN. It has been reported that KCN reacts with Cu to form [Cu(CN)_3_]^2−^, which usually occurs more easily than with Pb [61]. Although KCN is an effective agent to mask and diminish the effect of Cu, it is not recommended for use in food analysis because of its high toxicity. Alternative masking reagents for the Cu should be further investigated such as thiosemicarbazide and its derivatives [62,63] and potassium iodine [64]. In addition to Cu, other ions such as Al^3+^, Cr^3+^, or Fe^3+^ could interfere with the assay as those ions can potentially bind to the CA [38]. Among those ions, only Fe^3+^ is possibly found in a very small amount in egg white [65]. Nevertheless, common iron masking agents such as NaF [66,67,68] can be used to reduce the interference effect by Fe^3+^ and achieve better specificity.

The applicability of the proposed dPAD for testing Pb in drinking water is very promising, because the color distance reduced proportionally to the increase in Pb concentrations. However, the sensitivity of the dPAD was still not sufficient for implementation in real drinking water samples, as it needs a detection limit as low as 0.01 µg·mL^−1^ and 0.5 µg·mL^−1^ for beverages in a sealed container [69]. There is a report of using polyvinyl alcohol-capped silver nanoparticles to enhance the sensitivity of paper-based assays of Pb in water. This colorimetric detection accomplished the detection range of 0.02–1.0 µg·mL^−1^, albeit with the assistance of Image J analysis [12]. For real food sample matrices, the color reduction from urease inhibition by Pb was demonstrated in water and milk. The sensitivity of the method was approximately 8.0 µg·mL^−1^, which is a similar level to our proposed method, and it still did not meet the FDA regulation [5,69].

To follow the FDA regulation, we implemented a standard addition technique to facilitate the usage of the dPAD for the screening of Pb in real food samples. Nevertheless, our device can detect Pb at the 2.0 µg·mL^−1^ level; therefore, the dPAD combined with standard addition is very promising as an alternative tool for the low-cost and simple detection of Pb in food matrices, particularly in developing countries.

The standard addition technique helps the interpretation of results, especially when there is existing interference from complex sample matrices. In addition, it facilitates the measurement when the concentration of analytes is likely to be low. In this study, a series of concentrations of standard Pb were used for each measurement. Because various concentrations of Pb were spiked in the tested sample, different color distances were expected to be observed on the dPAD. The amount of Pb existing in food samples can be estimated by drawing a straight line connecting each detection channel, a concept that is similar to that previously reported for albumin index evaluation [41]. When applying it to food matrices, it seems that the viscosity effects of food fluids influence the flow on a dPAD, in which a higher viscosity results in a slower rate [70], consequently affecting the color distance [71]. In our study, the supernatant obtained from the century egg extractions was rather viscous, which might have affected the color distance formation. However, because of the use of the standard addition method, the variation in the sample viscosity could be diminished. In this study, using the standard addition method combined with drawing a straight line was effective for the screening of Pb at a cutoff level of 2.0 µg·mL^−1^. Our proposed method was superior to that of commercial rapid test strips for Pb screening. The color change observed on commercial strips was rather difficult to interpret because of the interference of the color background of the paper. Moreover, the color change of different concentrations was not obvious. Generally, distance-based detection is advantageous over the colorimetric method, because the bias from individual color perceptions can be diminished.

## 5. Conclusions

The developed dPAD proposed in this study provides superior advantages in terms of simplicity, rapidity, and affordability for screening the contamination of Pb in century eggs. Thanks to the strong binding between PEI and CA, the length of the red color deposition on the flow channel could be clearly observed, and it referred to the Pb content in the tested samples. Additionally, the proposed device is very promising for food safety screening, particularly in developing countries. In addition to its affordability and portability, the method also meets the requirements of using fewer chemical reagents, and it is more environmentally friendly in comparison to the use of other chelating compounds. Because of the visual detection of the color distance color formation and the simplicity of drawing a curve to estimate the lead content, extra instruments for color intensity analysis are no longer required. Distance-based visualization combined with the standard addition technique was shown to be a simple and effective method for the screening of Pb in food matrices because it can accurately determine Pb at the 2.0 µg·mL^−1^ cutoff level, which is the safety-regulated limit in many countries. Moreover, the dPAD can be performed at neutral pH levels; therefore, it has the potential for broad applications in different fields, such as environmental and biological samples. Nevertheless, the sensitivity of this platform needs to be further improved for widespread implementation in other food matrices or biological samples. Multifactorial optimization and appropriate pre-concentration of the samples could also probably solve this limitation.

## Figures and Tables

**Table 1:** Interference ratio of foreign ions.

Sample	dPAD
Tested foreign ions	Interference ratio of other metal ions to Pb
Cu	1
Ca	>20
Ni	>20
Zn	10
Mg	>20

**Table 2:** Determination of Pb in blinded spiked century eggs on the dPAD compared to the concentrations measured by atomic absorption spectroscopy (AAS).

Sample	dPAD (µg·mL^−1^)	AAS (µg·mL^−1^)
Sample 1	≥2	2.8916
Sample 2	≥2	2.8643
Sample 3	≥2	1.9568
Sample 4	≥2	2.8728
Sample 5	≥2	2.0063
Sample 6	≥2	2.0012
Sample 7	<2	0.0803
Sample 8	<2	0.0923
Sample 9	≥2	2.8950

## Data Availability

No new data were created or analyzed in this study. Data sharing is not applicable to this article.

## Supplementary Materials

The following are available online at https://www.mdpi.com/2079-6374/11/3/90/s1. Figure S1: The proportional relationship between the color distance with the carminic acid (CA) concentration on the polyethyleneimine (PEI)-immobilized distance paper-based analytical device (dPAD) using PEI at the concentrations of 0.02% and 0.05% (*n* = 3); Figure S2: The effect of pH on the color distance observed on the PEI-immobilized dPAD with 0.02% PEI; Figure S3: The effect of Cu on the measurement of Pb in the presence and absence of 5% potassium cyanide (KCN) (*n* = 3); Figure S4: The absorption spectrum shift of CA by means of the addition of Pb in 0.1 M of HEPES buffer (pH 7.0).
