# Supplementary material for: A Simple Distance Paper-Based Analytical Device for the Screening of Lead in Food Matrices
Source: Biosensors (Basel). 2021 Mar 22;11(3):90. doi: 10.3390/bios11030090 (PMC8004165; doi:10.3390/bios11030090)
Supplement: Supplementary file 1 [file biosensors-11-00090-s001.pdf]

Supplementary information

A simple distance paper-based analytical device for the screening of lead in food matrices

Kasinee Katelakha [1], Vanida Nopponpunth[2,3], Watcharee Boonlue[4,5] and Wanida Laiwattanapaisal[2,5,*]

1 Interdisciplinary Program of Biomedical Sciences, Graduate School, Chulalongkorn University, Bangkok, Thailand 10330
2 Department of Clinical Chemistry, Faculty of Allied Health Sciences, Chulalongkorn University, Bangkok, Thailand 10330
3 The Halal Science Center, Chulalongkorn University, Bangkok, Thailand 10330
4 Department of Nutrition and Dietetics, Faculty of Allied Health Sciences, Chulalongkorn University, Bangkok, Thailand 10330
5 Biosensors and Bioanalytical Technology for Cells and Innovative Testing Device Research Unit, Chulalongkorn University, Bangkok, Thailand 10330
* Correspondence: Wanida.L@chula.ac.th

PEI concentration optimization for immobilization on the dPAD

To determine the appropriate concentration of PEI for the distance measurement on the dPAD, 7.5 μL of PEI at the concentrations of 0.02% and 0.05% was deposited in the hydrophilic channel of the dPAD. Various CA concentrations (0.0–2.0 mmolL^{-1}) were tested. The results demonstrate that the color distance on the dPAD increased when the CA concentrations increased (Figure S1).

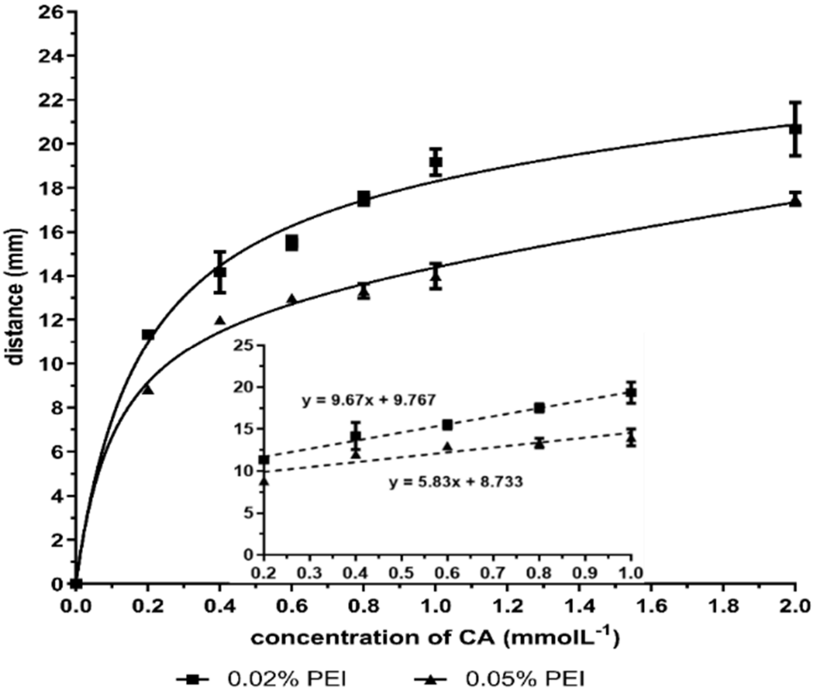

Figure S1. The proportional relationship between the color distance with the carminic acid (CA) concentration on the polyethyleneimine (PEI)-immobilized distance paper-based analytical device (dPAD) using PEI at the concentrations of 0.02% and 0.05% *(n = 3)*. Inset: Linear regression analysis and the best-fit line of CA between the ranges of 0.2 and 1.0 mmolL^{-1}.

Effect of pH on the color distance of CA on the PEI-immobilized dPAD

The effect of pH on the adsorption behavior of CA on the PEI-immobilized membrane was investigated using various types of buffers and pH levels. The buffer solution, including

sodium acetate buffer (pH 6.0), HEPES buffer (pH 6.0 and 7.0), sodium phosphate buffer (pH 6.0, 7.0, and 8.0), and tris-HCl (pH 8.0 and 9.0), was tested with 0.8 mmolL^{-1} of CA (Figure S2).

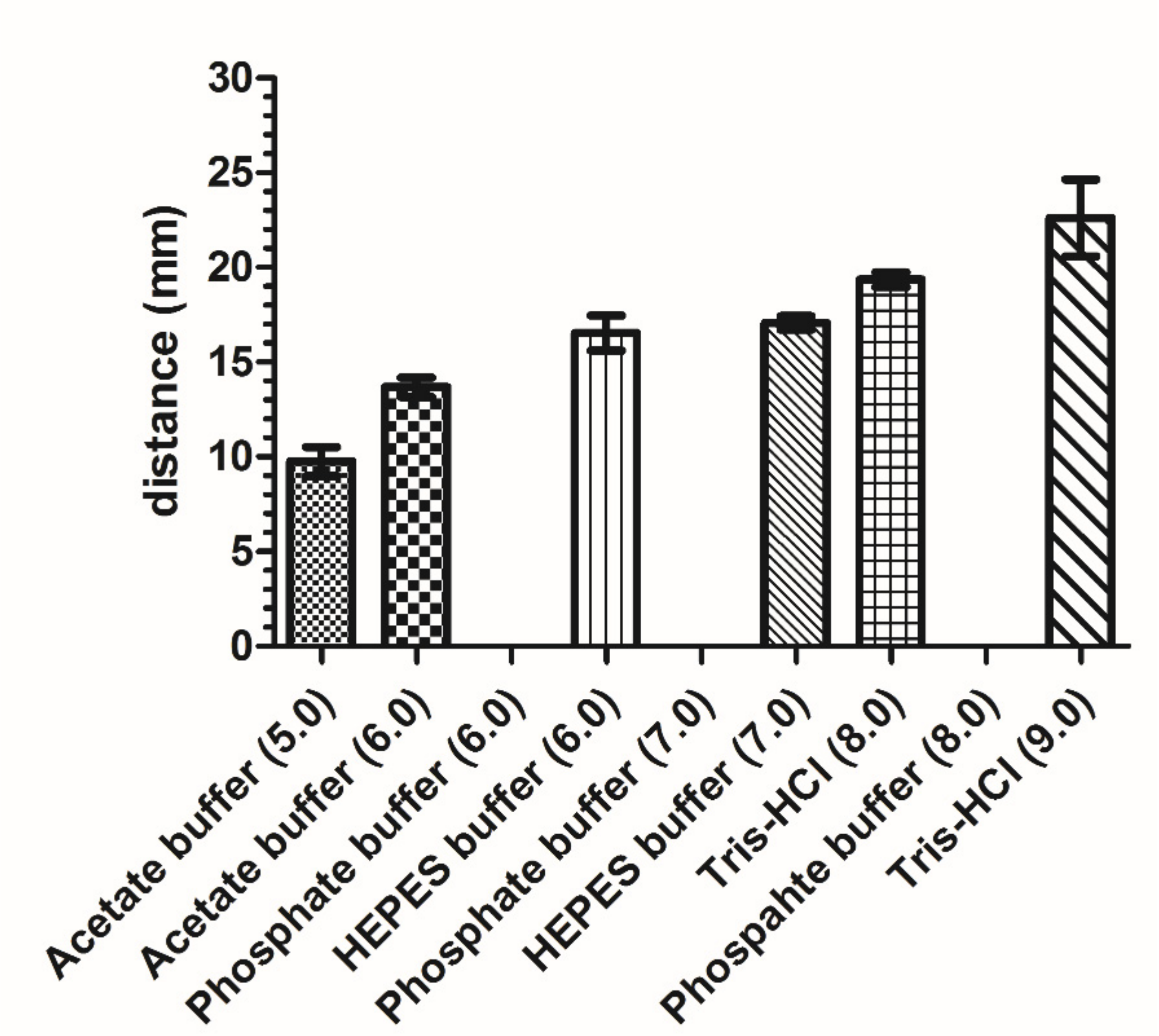

Figure S2. The effect of pH on the color distance observed on the PEI-immobilized dPAD with 0.02% PEI. All buffers were prepared at the concentration of 0.1 M. CA was prepared at the concentration of 0.8 mmoL^{-1} *(n = 3)*.

Effect of copper on the distance observed with the dPAD

The detection of Pb on the dPAD was affected by copper (Cu). In this study, the solution mixture was prepared with CA and a mixture of 20 μgmL^{-1} of Pb and 20 μgmL^{-1} of Cu. The distance was measured and it was found to be affected by Cu by a color distance reduction of

>5%. To demonstrate that the effect of Cu could be eliminated by KCN, KCN was added to the solution of CA and, the solution mixture of Pb and Cu was then added into the CA solution. The reduction of the distance is shown in Figure S3.

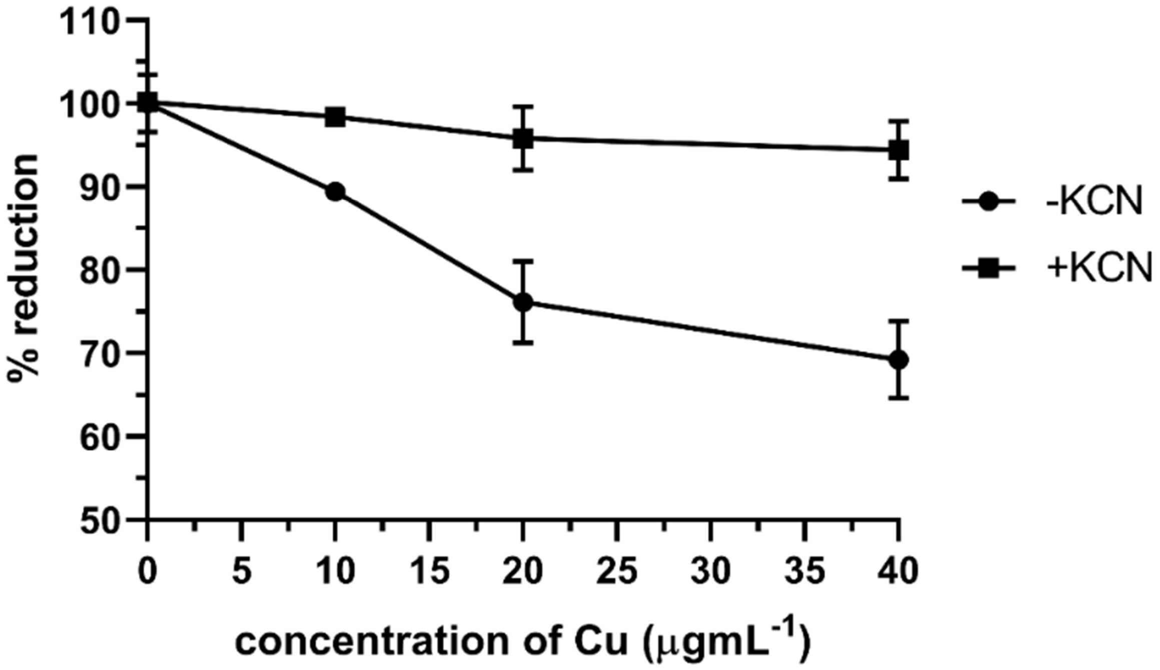

Figure S3. The effect of Cu on the measurement of Pb in the presence and absence of 5% potassium cyanide (KCN) *(n = 3)*.

CA–PEI complexation

The complex formation between CA and PEI was confirmed in batch solution by determination of the absorption spectrum shift in 0.1 M HEPES buffer (pH 7.0). The maximum absorption spectrum shift from 514 to 566 nm of CA exhibited an interaction of CA and PEI, as seen in Figure S4.

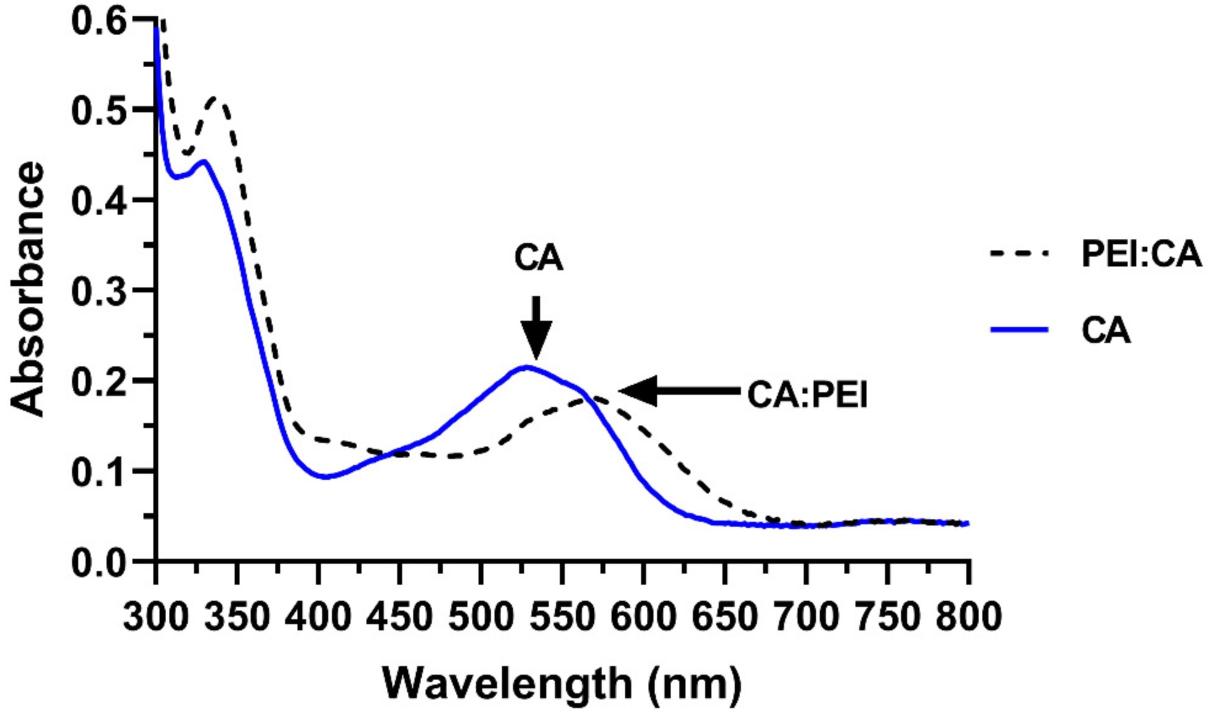

Figure S4. The absorption spectrum shift of CA by means of the addition of Pb in 0.1 M of HEPES buffer (pH 7.0).
